# Annotations

**Published:** 1905-05-13

**Authors:** 


### ANNOTATIONS.

### Early Open-Air Treatment of Phthisis Pulmonalis.

Dr. James Finlayson, for many years the Honorary Librarian of the Glasgow Faculty of Physicians and Surgeons, has recently discovered among the papers of the late Dr. Mackenzie, the distinguished ophthalmologist, a record of much interest in relation to the treatment of phthisis pulmonalis some hundred years ago. The document is the relation in detail by a patient of a system of treatment instituted and carried on by the Rev. Andrew Stewart, a doctor of medicine and also a parish minister. The starting point of this method is referred to the year 1796, and it is of interest to observe how in several points it anticipated certain modern developments in the treatment of tuberculosis. The "old system" is characterised as "a system of palliation," which by the fear of taking cold makes the patient "a complete hothouse plant, and implies a state of constant bondage." Hence patients were recommended to be for many hours daily in the open air, either on horseback or on foot, or in an open carriage, with a view to restore "the frame to its original healthy tone of vigour." The dieting scheme did not rival some recent efforts in the art of "stuffing," but it included a "moderate quantity of refreshing food," and was reinforced by "those early and regular hours which are most efficacious to digestion." The other feature of the treatment was the use of vinegar and water as an embrocation to the chest, throat, and limbs, to be followed by vigorous rubbing with flannel or a flesh brush. To the virtue of these methods the writer of the document in question offers grateful testimony and announces that he has not "caught cold" in spite of "most ungenial weather with constant east wind." The reverend inventor of this early open-air treatment, however, does not appear to have secured the confidence of his *confrères*, as in the records of a contemporary medical society he is described as "A Medico-Clerical Gentleman of Consumptive Notoriety."

### Plague in India and China.

In view of the alarming and portentous statistics of the plague epidemic in India, where there were 65,789 cases and 57,702 deaths for the week ended April 1st last, it is interesting to learn that the disease has this year been only sporadic in Hongkong, where the deaths from January 1st to April 1st only numbered 24. It is true that there were seven deaths in the week ending last Saturday; but the latest weekly returns for Calcutta give no fewer than 712 deaths; for the Bombay district 3,056; for the Madras district 81; for the Bengal district 9,703; and for the Punjaub 19,015. The deaths in the whole of India from plague numbered over one million in both 1903 and 1904, and it seems probable that 1905 will surpass their record. In Hongkong in 1904 the plague visitation was a very mild one, though the mere fact of its existence both then and now has led to the port being generally quarantined. It is satisfactory, however, to find that the great efforts made to improve the sanitation of the colony and to prevent the spread of infection have been attended with some success. The Chinese are fortunately less difficult to deal with than the Hindoos, there being an absence of caste prejudices among them, and though they are very strongly opposed to house-to-house inspection by foreigners, they are not unwilling to aid in taking measures of precaution and to allow cleansing operations to be carried on in their houses. They recognise to a certain extent what the Indians, apparently, are unable to do at all, that efforts must be made to check the spread of the malady. Like all Asiatics, the Chinese usually succumb to the plague, about 90 per cent. of the cases among them proving fatal, and they are anxious both on that account and because of the great interference it causes to trade, to see it stamped out. So far the Indian Government seems to have failed in impressing the natives with the paramount necessity of taking precautionary measures to prevent infection, which is obviously the first step towards imposing any check upon this scourge in Asia.

### Pathologists and Fees.

The Ethical Committee of the British Medical Association has acted well within its province in reviewing the action of certain practitioners, more or less well known as pathological experts, who have intimated their readiness to act in response to a coroner's summons to make a post-mortem examination and to give evidence at an inquest for a fee of two guineas. That fee is the statutory sum fixed for such services when they are rendered by a practitioner who has seen the person, whose death is the subject of inquiry, during life, or, failing such a witness, by any neighbouring practitioner. The general medical practitioner is not, nor does he pretend to be, an authority upon the more unusual and recondite problems of pathology, but he is perfectly fitted—and the law recognises this to be the case—to determine the cause of death in the great majority of the instances which come within the scope of a coroner's jurisdiction. The legal fee for such service to the public interest is two guineas, and it seems extraordinary that a number of gentlemen who claim to be skilled pathologists should express their willingness to contribute their special experience for the same fee. The result of such a course must inevitably be either a reduction of the fee in cases where the general practitioner is concerned or the employment of the expert at the expense of the practitioner. Opinions may differ whether the latter result is or is not desirable, but in any event it should be attempted through a clear issue and not by a side-wind. The action of the "experts" in question is wanting, as we think, in respect to the position they claim to occupy as well as in consideration for other members of the profession. Hence we are pleased to observe that it has attracted formal notice and has received a well-merited rebuke. There can be no doubt that the constituency which the Ethical Committee represents will emphatically endorse the action of the Committee, and it is to be hoped that the practitioners concerned will reconsider their position.

